# Is present pharmacy education adapted to needs? Survey results from young practitioner views regarding pharmacy education outcome towards a national reform in Hungary

**DOI:** 10.1016/j.jsps.2021.12.015

**Published:** 2021-12-31

**Authors:** András Fittler, Gabriella Nagy, Karina M. Füstös, Róbert Gy. Vida, Gábor Rébék-Nagy, István Szabó, István G. Télessy

**Affiliations:** a Department of Pharmaceutics, Faculty of Pharmacy, University of Pécs, Hungary; b Department of Languages for Biomedical Purposes and Communication, University of Pécs Medical School, Hungary; c Quality Management and Institutional Development Department, University of Pécs Medical School, Hungary

**Keywords:** Needs-oriented curriculum, Pharmacy education, Survey, Young pharmacists

## Abstract

**Background:**

The pharmacist career is constantly adapting to societal and health care needs. The past decade has seen a growing demand for curricular development to align graduation outcome with workforce competencies.

**Objective:**

This study aims to identify expectations for both didactic and experiential components of a new curriculum based on young pharmacist practitioner views.

**Methods:**

An online survey questionnaire was used in 2019–2020 to evaluate the pharmacy curriculum to detect indicators or key areas which require comprehensive reform.

**Results:**

The predominant majority of the 205 study participants recommended reduction in credit hours for Natural Sciences (78.54%) and a similar increase in the Theoretical and Practical Expertise Module (77.9%). Pharmaceutical care, clinical therapeutics and clinical pharmacy competencies should also be more highlighted in the program. Findings indicate the current training does not prepare for problem-solving and daily workplace challenges (72.7%) or for extended pharmacist skills and competencies (71.71%). Results show inconsistency in practical training experience, as all respondents participated in practical training for drug manufacturing and analysis but 61.0% reported no hands-on skills training in a hospital-clinical simulation setting. Indications for practitioner involvement into the natural sciences and biomedical subjects (86.3%) confirm the obvious need for more practice-oriented education.

**Conclusions:**

Educational reforms seem to be inevitable to achieve measurable improvement in professional practice and skills competency. The country specific demand for a needs-based pharmacy education reflects global trends but may also provide useful insights for individual transitions to transform education through practice and improve practice through education.

## 1 Introduction

The pharmacist profession needs to recognize and adapt to the market-oriented changes also affecting other health sectors today. Pharmacy education through strategically planned curricula must provide educational outcomes that serve ever-changing workforce needs and allow flexible adaptation to advancements in pharmacy (Arakawa et al., 2020). To update national undergraduate pharmacy education and training program, a comprehensive and clear view of both present and future goals is needed in higher education institutions (HEI) to optimize the strategic planning and decision-making process of a curricular development. For a better understanding of the context, we propose to address the issue from three different perspectives, (a) The *international recommendations upon professional development and pharmacist education* by the International Pharmaceutical Federation (FIP) Development Goals framework (International Pharmaceutical Federation (FIP), 2020), the Pharmacy Education in Europe (PHARMINE) project (Atkinson and Rombaut, 2011) and the Quality Assurance in European Pharmacy Education and Training (PHAR-QA) (Atkinson, 2017), (b) The *national changes required in pharmacy education,* Hungarian professional organizations have been encouraging during the past decade (Botz et al., 2015, Fittler et al., 2014, Hungarian Chamber of Pharmacists, 2015) and, (c) *graduates’ perceptions of* pharmacy education. The goal of this study is to explore the process of change and identify relevant objectives through the interpretation of novice Hungarian pharmacists’ views and to make prospective recommendations for updating pharmacy education in Hungary.

**Fig. 1:**
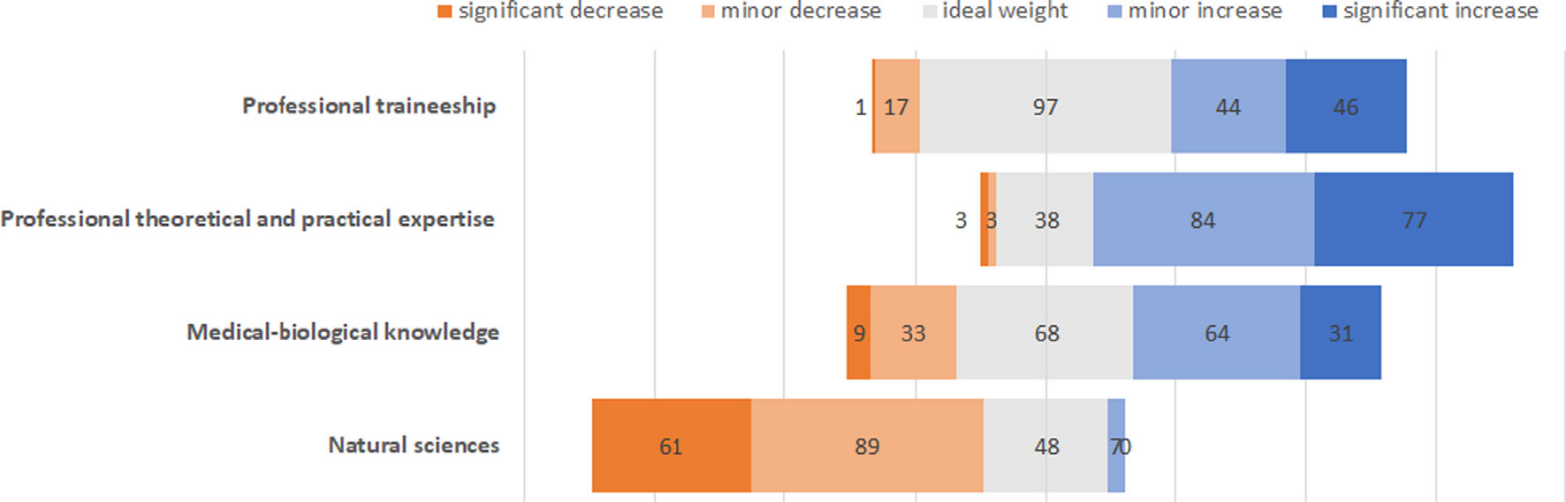
Recommended changes in credit hours of educational modules. Data are presented on a diverging stacked bar with a neutral split (grey area for ideal weight) indicating no preference for change. The Natural Sciences Module includes general, inorganic and organic chemistry; mathematics and biostatistics; the Biomedical Module incorporates anatomy; microbiology; biology and biochemistry; physiology, pathology, and public health, while the Professional Theoretical and Practical Expertise Module includes pharmaceutical technology, pharmaceutical chemistry, social- and administrative pharmacy, and pharmacology.

The FIP document (Pharmaceutical Workforce Development Goals) of September 2020 provides the latest guidelines for pharmacy education development both at a national and global levels by setting out the goals, requirements and recommendations, also allowing flexibility for country-specific implementation plans (International Pharmaceutical Federation (FIP), 2020). The FIP document clearly advocates a needs-based transformation for all areas of pharmacy including practice, science, education and workforce. As a precursor, the first PHARMINE project mapped out European pharmacy education in 2011, publishing 25 country profiles of structure and competencies (Atkinson and Rombaut, 2011). The results showed heterogenous pharmacy education in Europe with very limited data on competency-based learning (Atkinson, 2014). As a PHARMINE follow-up, the PHAR-QA directives proposed a more detailed competency framework for pharmacy education, providing an opportunity for HEIs to create competency-based curricula or to initiate curricular outcome alignment with the framework guidelines (Atkinson, 2017, Atkinson et al., 2016, Atkinson and Rombaut, 2011). However, as of today, only minor changes were implemented in the Hungarian pharmacy education programs. For a better understanding of the national pharmacy education structure and background, see Supplementary file 1**.**

To highlight expectations regarding curricular content, teaching methodology, usefulness and employability, the present study aims to provide an evaluation of the current Hungarian pharmacy training and education system based on the reflective views of a representative sample of recently graduated pharmacist practitioners.

## 2 Methods

Initially, a national survey questionnaire from 2014 (Fittler et al., 2014) was adopted by the authors in 2019. The questionnaire was updated to integrate reports from the Hungarian Chamber of Pharmacists and the National Accreditation Committee issued in 2015 (Botz et al., 2015, Hungarian Chamber of Pharmacists, 2015). The questionnaire was assessed by five academic teacher practitioners to improve relevance, clarity and content validity. Content validity index has not been measured. The revised list of questions was piloted online (via Google Forms) by two recently graduated pharmacists from each HEI, in November 2019. No comments on the relevance or issues regarding the ease of understanding were made by the pilot participants. Subsequently, the 19-item national language anonymous questionnaire was distributed via the Hungarian Chamber of Pharmacists website and was available for three months, from November 2019 through January 2020. Recruitment of pharmacists was carried out by using a convenience sampling technique, no pre-determined sampling frame was used.

The first four questions explored the respondents’ field of work, place and date of graduation, Nos. 5–10 explored the perceived relative weight of each training module based on credit hour value and the form of teaching. The recommended changes in credit hours for a module or knowledge content were expressed using a five-point Likert scale (+2 = significant increase, +1 = minor increase, 0 = ideal weight, −1 = minor decrease, −2 = significant decrease). Respondents also evaluated the integration or exclusion of knowledge competencies taught. Questions 11–17, focused on the alignment between academic training and practice, attitudes toward undergraduate specialization, the opinions regarding the adequacy of teaching methodologies, integration of pharmaceutical practitioners into undergraduate education, and the institutional management and usefulness of traineeship experience. No. 18 offered space for subjective evaluation of the Alma Mater, and No. 19 for sharing additional remarks. However, introduction of the qualitative data exceeds the scope of this paper. The English translation of the survey is presented in Supplementary file 2**.**

The pharmacist professionals cohort sample included participants with a fresh pharmacist degree who volunteered to complete the survey. No participant identities were recorded and confidentiality was guaranteed. Due to the very nature of the survey, no ethical approval was necessary. Data analysis was performed using SPSS Statistics 26 for Windows.

## 3 Results

### 3.1 Characteristics of the study participants

During the three-month period, 261 responses were collected. Descriptive statistics and evaluation of the responses are presented in our current work for the target group of 205 young pharmacists graduating between 2010 and 2019. Participants responded from all HEIs of Hungary. The respondents graduated from Budapest (32.7%), Szeged (28.8%), Pécs (24.4%) and Debrecen (14.1%). All fields of pharmacy were represented, of which, community pharmacists comprised 63.4% of the total number of participants, while 15.1% of the responses came from hospital and clinical settings, 9.3% from academic staff in education and research, 8.3% from the pharmaceutical industry including research and development and 3.9% from other sectors such as wholesale, authorities. The average duration of employment of the respondents was four years (3.99 ± 2.46).

### 3.2 Responses on credit hour value regarding educational modules, subjects and required new areas of knowledge in the curriculum

Participants were asked to make recommendations for changes in the pharmacy program, with special regard to the credit hour value of the three educational modules and pharmacy traineeship See Fig. 1.

Substantial decrease in credit hours was recommended in the Natural Sciences Module by a significant majority (n = 150, 73.2%) of the respondents. At the same time, 161 respondents (77.9%) recommend a nearly identical increase in the Professional Theoretical and Practical Expertise Module. Every second respondent (n = 97, 47.3%) considered the relative weight of traineeship ideal, however, 90 respondents (41.6%) believe it is necessary to increase the time allocated to this module.

A closer analysis of the subjects in each module demonstrated a clearer picture regarding the recommended changes in credit values in the various fields of professional knowledge listed in the curriculum. Based on the relative weight of responses, the curriculum should be “significantly reduced” in areas of physical chemistry (43.4%), mathematics and biostatistics (28.3%), history of science and propedeutics (25.9%) and pharmaceutical chemistry (24.9%). At the same time the number of credit hours should be “significantly increased” for pharmaceutical care (41.9%), clinical pharmacotherapeutic studies (39.0%), clinical pharmacy (31.7%), pharmacology (30.2%), pathophysiology (24.4%), pharmaceutical management (20.5%), and for traineeship in industrial setting (23.9%). Teaching was considered ideal for pharmaceutical technology (55.1%), biology (61.5%), Latin language (64.4%) and pharmaceutical ethics (55.6%). Detailed results presenting the average value of change on a −2/+2 Likert scale in support of the subjects are presented in Appendix 1.

The respondents were also asked to name the form in which the core competencies should be integrated into our pharmacy education (as an individual obligatory or elective course, or integrated into an obligatory course, or if they should be omitted and not be taught at all). The highest response rates involved pharmaceutical care, clinical therapeutic studies, clinical pharmacy and informatics (70.7%, 45.4%, 45.4% and 32.7% respectively) which the respondents preferred to be offered as obligatory courses. Based on the percentage of the most frequent responses in our dataset, respondents recommended the integration of the following subject areas into existing obligatory courses: legal studies (56.6%), drug manufacturing (53.7%), authorization (52.2%), therapeutic appliances and medical devices (49.8%), pharmaceutical ethics (49.3%), quality assurance (48.8%), biopharmacy (46.8%), drug development (47.3%), health-sciences including prevention and dietetics (46.8%), toxicology (45.4%), biotechnology (42.4%), and professional communication (40.0%).

### 3.3 The relationship between training and daily practice and recommendations regarding the general management of training

Although the required theoretical knowledge tends to change, one premise remains unchanged: problems need to be solved. Accordingly, one of the key questions in training is whether it encourages the development of problem-solving skills. As many as 72.7% of the respondents share the opinion in which the current level of training does not adequately develop students’ problem-solving skills, nor for tackling daily challenges in the workplace. Upon further questioning it turned out that 71.71% of the survey participants did not concur today’s training recognizes the extended pharmacist skills and competencies of the 21st century. A definite need for practice-oriented teaching was reflected in an indicative result showing that 86.3% of the respondents believe the topics of the Natural Sciences and Biomedical module subjects clearly need development through the involvement of pharmacist practitioners. Only as few as 3.9% of the respondents did not agree with this statement.

Our study results show that nearly half of the respondents (43.9%) agreed that the present Hungarian training is still characterized by ‘drug-centeredness’. Interestingly, 33.3% showed indifference on this issue. At the same time, only one-fifth agreed partially (21.0%) or fully (3.4%) that current pharmacy education is characterized by ‘patient, disease, and therapy’ centeredness.

Although the Master’s degree program needs to prepare future pharmacists for various career pathways of the profession, the length of the training and number of contact hours is limited. For this reason, we surveyed participants’ opinions on the possibility for a specialization option which 47.3% fully agreed with, while 20.5% accepted the possibility of undergraduate specialization. An obvious solution for integrating new knowledge and skills seems to be the revision of curricular network and elimination of recurring contents. Although more than half of the study participants (57.56%) agreed that the course setup is consistent and creates an optimal network, a contradictory finding shows more than two-thirds (68.78%) of the participants found redundant elements throughout their training. 26.8% of the responses did not find the course setup consistent enough, which supports the need for structural curricular reform.

Eight educational techniques were evaluated by the respondents based on the preference and perceived conformity in pharmacy education on a 5-grade Likert scale (-2 indicating definitive preferred reduction in education, 0 for optimal weight, while +2 representing the need for significant increase). As shown in Table 1, lectures and laboratory practice seem to be less popular with colleagues who support the more frequent use of interactive and problem-oriented learning methods. Respondents expressed the strongest need for more case studies, simulation and situational activities and group projects, all of which will enhance soft skills development, such as ability to work independently or in a team.

**Table 1:** Preference and perceived conformity of educational techniques in pharmacy education by graduate pharmacists on a five-point Likert scale between values −2 and + 2 (n = 205).

Form of class and delivery of teaching	Average preference value	Std. Deviation
Lecture	−0.70	0.78
Laboratory practice	−0.32	0.97
Case discussion	+1.41	0.69
Simulation-based training	+1.22	0.85
Team assignment	+1.06	0.95
Individual assignment	+0.79	0.97
Seminar	+0.59	0.90
Student presentation	+0.37	1,03

We aimed to explore the use of six practical and hands-on skills developing techniques during pharmacy education using a three-grade scale: ‘not applied’, ‘occasionally’ and ‘regularly used’ during education. Majority of the respondents stated they regularly used a community pharmacy compounding (95.1%) skills lab. Based on our survey results, graduated pharmacists in Hungary regularly, or at least occasionally, participated in hands-on practice related to industrial drug manufacturing (95.2%), drug analysis and quality control (92.7%), to community pharmacy dispensing, counselling and pharmaceutical care (64.8%), and in the use of pharmacy management software (60.0%). On the other hand, 61.0% did not have the opportunity to participate in hands-on training in a hospital-clinical simulation environment (patient consultation, preparation of injectable medications).

### 3.4 Reflections on pharmacy traineeship

Aspects of usefulness and organization of traineeship were evaluated on a five-grade scale measuring the extent to which respondents agree with the statements. Respondents recognize traineeship as an integral part of pharmacy education, since most agree or strongly agree that the one-month regular traineeship (61.0%) and the final year traineeship (62.9%) are beneficial in their current form. A revision in the management and curricular update of pharmacy traineeship should be considered, as a representative portion (40.0%) of the respondents felt traineeship is not appropriately coordinated by the HEIs and 41.0% did not find curricular content or course descriptions properly defined. These anticipated changes will also be beneficial to improve student integration into traineeship site workflow, since only one-third of the responding pharmacists agreed or strongly agreed (35.1%, 24.4% respectively) in which second and third year students provide a valuable workforce. Responses regarding different traineeship settings presented in Table 2**.** clearly illustrate the need for pharmacy traineeship in hospital, clinical and industrial settings.

**Table 2:** Attitudes of novice pharmacists regarding different traineeship settings.

Statement	Rate of agreement N (%)				
	strongly disagree	disagree	neutral	agree	strongly agree
The length of community pharmacy traineeship is sufficient.	6 (2.9%)	26 (12.7%)	22 (10.7%)	71 (34.6%)	80 (39.0%)
The length of hospital-clinical traineeship is sufficient.	47 (22.9%	71 (34.6%)	31 (15.1%)	37 (18.0%)	19 (9.3%)
Students gain sufficient clinical knowledge (e.g., during hospital visits) during traineeships.	103 (50.2%)	67 (32.7%)	20 (9.8%)	11 (5.4%)	4 (2.0%)
Students spend sufficient traineeship time in the pharmaceutical industry.	118 (57.6%)	52 (25.4%)	22 (10.7%)	7 (3.4%)	6 (2.9%)

## 4 Discussion

In our national survey among early career pharmacists, we generated invaluable needs-based evidence and feedback regarding the recommended credit hour values for educational modules and knowledge areas which should be taught more or less extensively. We identified a required shift in the educational focus, since recently graduated pharmacists welcome a substantial decrease in credit hours within the Natural Sciences Module and suggested a similar increase within the Professional Theoretical and Practical Expertise Module. Aligned with the international professional development goals, educational content should shift from ‘product-, medication- and manufacturing- centered’ curricula to ‘patient and therapy-centered education’ (Nunes-da-Cunha et al., 2016). Our national survey findings on the recommended reallocation of credit hour values correspond with the European shift in the ‘medical sciences’/’chemical sciences’ courses credit value ratio described by Atkinson (2014). Similarly, pharmacist or pharmacy student perception studies showed that not only in Europe, but also in the Eastern Mediterranean Region there is a general need for more practice-based training and modernization of the curriculum. Perception surveys conducted in Egypt, Sudan, Qatar, Kuwait, Pakistan and Saudi Arabia regarding undergraduate education showed similar results, research participants were not satisfied with the curriculum, stating it is outdated and has minimal relation to the everyday pharmacy practice (indicating the inclusion of CAM therapy knowledge and clinical skills, pharmaco-economics, or social pharmacy) (Bajis et al., 2016). The work of Arakawa et al showed that the chemistry-centered curriculum and the recommended changes highlighted in our study are also present in Bosnia and Herzegovina, Croatia, Austria and Iceland (Arakawa et al., 2020).

The overwhelming majority of Hungarian early career pharmacists as survey participants shared the opinion that their training did not develop student problem-solving skills, nor did it provide adequate preparation for the extended pharmacist skills and competencies of the 21st century. In line with international publications on the usefulness of educational activities, it is essential to select methods which allow close focus upon the development of individual problem-solving including teamwork skills, and to integrate practice-oriented methods for a better understanding of the syllabus (Gustafsson et al., 2021, Katoue and Schwinghammer, 2020). The focus of instruction and educational techniques needs to shift from affirmation of lexical knowledge via lectures towards the flexible application of referential knowledge transfer, such as case-discussions and simulation-based learning methods.

Specialized skills laboratories within the university, research and thesis projects, and especially the final-year traineeship offer feasible solutions and provide adequate opportunity to acquire specialized professional skills and knowledge within the five-year pharmacy education system. Survey respondents highlighted the lack of opportunities to participate in hands-on training in hospital-clinical environment offered within the training institutions. Further, a markedly high percentage of our survey participants believe the topics of the Natural Sciences and Biomedical Module subjects should be designed with the involvement of pharmacist practitioners.

The study has several strengths and, admittedly, certain limitations. As the focus of the survey was upon the young pharmacists who recently received their pharmacist degree and gained fresh experience in contemporary pharmacy practice, we can assume their reflections are valid regarding the current curricula and majority of the respondents was given the opportunity to recognize the usefulness of the acquired knowledge and skills necessary to meet daily challenges. We consider our study cohort representative for young pharmacist professionals as the completion of the questionnaire is 5.9% of the total number of newly registered pharmacists in the period of 2010–19. We see only a minor limitation that more than half of the respondents were from the community pharmacy sector and the findings mainly reflect community pharmacists’ preferences. As limited published data is available regarding national pharmacy education and training, our findings and the country profile available in Supplementary file 1. resolves the knowledge deficit for pharmacist education in Hungary.

During the past decades, despite the diversity within the profession, medication- and distribution-centered pharmaceutical practice has gradually transitioned into patient and therapy-oriented service in response to the global shift towards more personalized and patient-centered forms of health care (International Pharmaceutical Federation (FIP), 2020, Katoue and Schwinghammer, 2020, Nunes-da-Cunha et al., 2016). In addition to the traditional pharmacist roles and expectations regarding compounding, patient service and quality assurance, there is a growing tendency for the recognition of the improved cognitive pharmaceutical services. More complex and extended novel pharmacist competencies are being recognized far beyond merely dispensing and compounding (Volmer et al., 2019).

For better results, this paradigm shift needs to be succeeded by a similar shift in educational approach: the traditional ‘institution-centered’ approach should develop into a more ‘outcome-focused competency-based’ approach. Although traditional education systems focus on the teaching process and the instructor, current literature highlights the use of a competency-based approach in education, in which educators and students share responsibility for the learning process and its outcome (Katoue and Schwinghammer, 2020). This is aligned with the FIP goals emphasizing a collaborative learning model, in which students should be active participants, not passive recipients, during their education (International Pharmaceutical Federation (FIP), 2020, Katoue et al., 2014). It must be noted, such learning outcome orientation is a core principle of the European and National Qualifications Frameworks, supporting the transparency of education and training systems, thereby enhancing compatibility between countries and higher educational institutions within countries, including cross-border learner mobility support, for example, the Erasmus+ program.

## 5 Conclusions

The pharmacist career, including its opportunities and challenges, is constantly evolving, not only for the individual, but also for all fields of the profession. Ideally, changes are tailored and adapted to societal and health care needs. The national survey results presented in this study provide useful country-specific information received from practitioners on needs-assessment in education, as no assessment of pharmacy education in Hungary has been published before. It must be emphasized that the Hungarian survey results are consistent with the national and international expectations regarding pharmacist education, and this will hopefully encourage their successful implementation through the decisions of academic directors and professional bodies.

Fast-paced development in the pharmacy profession created a growing need for educational reforms, which clears the path for measurable improvements in professional practice and for competency development. Although the professional and educational development goals may be consistent, the means and modalities still vary within the EU and very likely, globally. Schools cannot replicate the curricula of other institutions since the educational facilities, human resources, financing, and institutional policies vastly differ. Accordingly, each pharmaceutical academic institution must find its own way of continuous development in transforming education through practice and improving practice through education.

## CRediT authorship contribution statement

**András Fittler:** Conceptualization, Methodology, Data curation, Investigation, Writing – original draft, Writing – review & editing, Supervision. **Gabriella Nagy:** Conceptualization, Writing – original draft, Writing – review & editing. **Karina M. Füstös:** Conceptualization, Methodology, Data curation, Investigation, Writing – original draft. **Róbert Gy. Vida:** Conceptualization, Methodology, Data curation, Writing – review & editing. **Gábor Rébék-Nagy:** Writing – review & editing. **István Szabó:** Writing – original draft, Statistics. **István G. Télessy:** Conceptualization, Writing – original draft, Writing – review & editing, Supervision.

## Declaration of Competing Interest

The authors declare that they have no known competing financial interests or personal relationships that could have appeared to influence the work reported in this paper.

## Figures and Tables

**Table:** 

Field of professional knowledge (subject title)	Average preference value	Std. Deviation
***Natural sciences module***		
Analytical chemistry	−0.73	0.8
General and inorganic chemistry	−0.58	0.80
Information technology	−0.02	1.21
Inorganic chemistry	−0.62	0.77
Mathematics. biostatistics	−0.84	0.94
Organic chemistry	−0.57	0.83
Physical chemistry	−1.18	0.81
***Medical-biological knowledge module***		
Anatomy	0.34	0.93
Biochemistry	−0.06	0.92
Biology	−0.13	0.77
Biophysics	−0.72	0.78
First Aid	0.49	0.89
Immunology	0.33	0.92
Latin language	−0.34	0.78
Microbiology	0.19	0.92
Pathophysiology	0.7	0.95
Pharmacobotany	−0.51	0.91
Physiology	0.5	0.83
Public Health	−0.23	0.9
***Professional theoretical and practical expertise module***		
Biopharmacy	0.21	0.79
Clinical Pharmacotherapy	1	0.93
Clinical Pharmacy	0.87	0.93
History of Pharmacy and Propedeutics	−0.8	0.82
Pharmaceutical Biotechnology	0.38	0.91
Pharmaceutical Care	1.1	0.87
Pharmaceutical Chemistry	−0.69	0.88
Pharmaceutical ethics	−0.02	0.87
Pharmaceutical Practice and Management	0.62	0.93
Pharmaceutical Technology	−0.01	0.88
Pharmacognosy	−0.3	0.96
Pharmacology	0.95	0.82
Pharmacopoeial Analysis	−0.27	0.85
***Traineeships***		
Summer pharmacy traineeships	0.18	0.85
Pharmaceutical industry traineeship	0.73	0.86
Pre-final community pharmacy traineeship	0.09	0.87
Pre-final hospital-clinical traineeship	0.39	0.86

## Appendix A Recommended changes for credit values of professional knowledge in Hungarian pharmacy education based on the responses of novice pharmacists on a five-point Likert scale between values −2 and +2 (n = 205)

Recommended change in credit hours (+2 = notable increase, +1 = minor increase, 0 = ideal weight, −1 = minor decrease, −2 = notable decrease) of a given module or knowledge are presented as average values and standard deviation.
