# Supplementary material for: Is present pharmacy education adapted to needs? Survey results from young practitioner views regarding pharmacy education outcome towards a national reform in Hungary
Source: Saudi Pharm J. 2021 Dec 31;30(2):132–7. doi: 10.1016/j.jsps.2021.12.015 (PMC9072685; doi:10.1016/j.jsps.2021.12.015)
Supplement: Supplementary data 1 [file mmc1.docx]

**Supplement 1.: Pharmacy education in Hungary**

The Hungarian pharmacy curriculum comprises a five-year (one-tier) Master's degree course with 300 ECTS (European Credit Transfer and Accumulation System) credits collected through a course-based system, and a six-month traineeship to be completed during the fifth academic year. Pharmacy education is provided at the university level in four Higher Education Institutions (HEIs) in the country, with traditional medical subject-based study programs and product-oriented curricula, aligned to the EU directive referencing sectoral professions providing the basis for recognition of qualification. The Hungarian Education and Learning outcome Requirements (ELR) identify student professional knowledge content and competency requirements, however, each training institution has the autonomy to determine the site and model for integrating the required professional knowledge.

Pharmacy students acquire learning competencies in three modules:

(1) **Natural Sciences Module** including general, inorganic and organic chemistry, mathematics and biostatistics, etc.;

(2) **Biomedical Module**: anatomy, microbiology, biology and biochemistry, physiology, pathology, public health, etc.; while the

(3) **Professional Theoretical and Practical Expertise Module** includes pharmaceutical technology, pharmaceutical chemistry, social- and administrative pharmacy, and pharmacology.

Clerkship offers essential experiential learning opportunity in undergraduate pharmacy education. Currently, pharmacy students in Hungary only have the opportunity to complete their final six-month pharmacy traineeship in a community and hospital pharmacy setting, while the one-month summer clerkship following the second and third years may also be completed in other fields of pharmacy practice.

Starting in 1999, clinical pharmacy and pharmaceutical care competencies have been gradually included and integrated into the faculty curricula representing a first step towards a more patient-oriented program. This was followed up with the introduction of additional educational content including pharmacist communication, problem-solving pharmacy or pharmaceutical counselling intending to open up new vistas to service-oriented realms in pharmacy. Admittedly, in the past decade, there has been an increasing demand for curricular development.

In 2014 a national report was published by the Hungarian Society for Pharmaceutical Sciences Committee of Youth [4], which revealed startling survey results based on the responses from 449 licensed pharmacists. In a national context, the most relevant expectations were formulated by the Hungarian Chamber of Pharmacists in 2015. Additionally, the need for changes in Hungarian pharmacy education were also highlighted by the Hungarian Accreditation Committee in 2015. Although these documents urged changes in Hungarian pharmacy education, only limited advancement was observed at the national level, the result being only minor curricular changes effectively implemented throughout the pharmacy education.

Long awaited revision and renewal of national and European legislation has recently been started. comprehensive and thorough revision of the national Education and Learning Outcome Requirements (ELR) was inevitable to improve and harmonization of pharmacy training curricula within and between the Hungarian pharmacy schools. Change was further triggered by the need for the national reform detailed above and the requirements set by national organizations.

The ELR approved by the National Education Authority serves as the legal basis of accreditation among HEIs through which universities are required to renew their operational licence every five years. The ELR describes pharmacists’ acquired learning competency domains (knowledge, skills, attitudes and autonomy), furthermore, it assigns the credit values to the three educational modules: knowledge in (natural sciences, biomedical knowledge, and professional theoretical and practical expertise) [8].

The Ministry of Innovation and Technology initiated the renewal process in the last months of 2020 and the new legislation regarding ELR expected is likely to come into force in 2021. Such national reform may clear the path for changes in the curriculum. We do hope the nationwide reform will soon be accompanied by mapping and assessment in reference to the development for sectoral professions under the 2005/36/EC directive, issued by the European Parliament and Council, articulating the recognition of professional qualifications. Unfortunately, the articles regarding the EU directive in aspects of training and pursuit of professional activities fail to provide complete coverage for either the present or the newly developing pharmacy roles to effectively assist pharmacists in meeting them, or exceeding, the professional, societal and stakeholders expectations. It is not surprizing, considering the fact that the currently valid minimum training requirements for pharmacists were originally formulated in 1985.

References

Ministry of Human Capacities Decree 18/2016 (VIII. 5.) on the Learning Outcomes of HE Vocational Trainings, BA and MA Programmes and on the Modification of the Ministry of Human Capacities Decree 8/2013. (I. 30.) on the Common Requirements of Initial Teacher Education and the Learning Outcomes of Each Initial Teacher Education Programme. Available online at: https://net.jogtar.hu/jogszabaly?docid=A1600018.EMM&txtreferer=00000001.txt&pagenum=4 (accessed July 2021)

Directive 2005/36/EC of the European Parliament and of the Council of 7 September 2005 on the recognition of professional qualifications. Available online at: https://eur-lex.europa.eu/legal-content/EN/TXT/?uri=celex%3A32005L0036 (accessed July 2021)

Fortuit P. Synthesis of the national experience reports on the directive 2005/36/EC relative to the recognition of qualifications for pharmacists. 2010. Available online at: https://ec.europa.eu/docsroom/documents/15384/attachments/8/translations/en/renditions/pdf (accessed July 2021)
